# Transient drought during flowering modifies the grain proteome of bread winter wheat

**DOI:** 10.3389/fpls.2023.1181834

**Published:** 2023-06-27

**Authors:** Olha Lakhneko, Oleg Stasik, Ľudovit Škultéty, Dmytro Kiriziy, Oksana Sokolovska-Sergiienko, Mariia Kovalenko, Maksym Danchenko

**Affiliations:** ^1^ Institute of Cell Biology and Genetic Engineering, National Academy of Sciences of Ukraine, Kyiv, Ukraine; ^2^ Institute of Plant Genetics and Biotechnology, Plant Science Biodiversity Centre, Slovak Academy of Sciences, Nitra, Slovakia; ^3^ Institute of Plant Physiology and Genetics, National Academy of Sciences of Ukraine, Kyiv, Ukraine; ^4^ Institute of Virology, Biomedical Research Centre, Slovak Academy of Sciences, Bratislava, Slovakia; ^5^ Educational and Scientific Centre (ESC) “Institute of Biology and Medicine”, Taras Shevchenko National University of Kyiv, Kyiv, Ukraine

**Keywords:** *Triticum aestivum*, water shortage, photosynthesis, contrasting cultivars, oxidative stress, yield quality, potential tolerance markers

## Abstract

Drought is among the most limiting factors for sustainable agricultural production. Water shortage at the onset of flowering severely affects the quality and quantity of grain yield of bread wheat (*Triticum aestivum*). Herein, we measured oxidative stress and photosynthesis-related parameters upon applying transient drought on contrasting wheat cultivars at the flowering stage of ontogenesis. The sensitive cultivar (Darunok Podillia) showed ineffective water management and a more severe decline in photosynthesis. Apparently, the tolerant genotype (Odeska 267) used photorespiration to dissipate excessive light energy. The tolerant cultivar sooner induced superoxide dismutase and showed less inhibited photosynthesis. Such a protective effect resulted in less affected yield and spectrum of seed proteome. The tolerant cultivar had a more stable gluten profile, which defines bread-making quality, upon drought. Water deficit caused the accumulation of medically relevant proteins: (i) components of gluten in the sensitive cultivar and (ii) metabolic proteins in the tolerant cultivar. We propose specific proteins for further exploration as potential markers of drought tolerance for guiding efficient breeding: thaumatin-like protein, 14-3-3 protein, peroxiredoxins, peroxidase, FBD domain protein, and Ap2/ERF plus B3 domain protein.

## 1 Introduction

Global warming increases the frequencies of mild and severity of extreme drought events (Kim et al., 2020). Such climate change negatively affects the yield of crops in many regions of the world (FAO, 2015). Drought causes a reduction in crop productivity due to a shortage of water availability, for example, from precipitation and quick evaporation from the soil, affecting food security worldwide (Wilhite and Glantz, 1985; Karatayev et al., 2022). Projecting the economic impact of climate change, experts estimated that annual losses in agriculture from drought are between 3.5–5.4 billion € within European Union and the United Kingdom (European Commission, Joint Research Centre et al., 2020). The severity of its influence depends on the duration of the exposure to drought and the stage of plant development (Morgun et al., 2019; Wan et al., 2022).

Under drought conditions, plants experience changes on different levels: molecular, biochemical, physiological, and morphological. Primary, drought promotes water loss in plants. Abscisic acid-mediated mechanisms, such as stomata closure, are typically activated to avoid desiccation. However, this leads to a decline in CO_2_ intake. Limited availability of CO_2_ reduces photosynthesis activity by causing an imbalance between light and dark reactions, promoting the superoxide radicals formation. The level of injury depends on plant tolerance. Simultaneously, activated photorespiration generates about 70% more H_2_O_2_ under restricted water conditions (Wingler et al., 1999). Such accumulation of reactive oxygen species (ROS) damages photosynthetic apparatus (Bashir et al., 2021). To overcome oxidative stress, a plant activates an enzymatic antioxidant system (superoxide dismutases, catalases, and peroxidases). Antioxidative enzymes scavenge ROS and thus restrain lipid peroxidation (Bashir et al., 2021; Seleiman et al., 2021).

Bread wheat (*Triticum aestivum*) is a worldwide consumed cereal, with an annual production of around 770 million tonnes (FAO, 2022). It is an allohexaploid (AABBDD, 2n = 6x = 42) species (Li et al., 2015). The main component of its grains is starch (about 80% of dry weight). Protein composition can vary broadly, from 7 to 22% on a dry weight basis depending on genotype (Vogel et al., 1976; Shewry et al., 2013). Storage gluten fraction (glutenins and gliadins) comprises the main part of the grain proteome and makes up to 70-80% of the total flour protein (Altenbach, 2017). Glutenins and gliadins define the bread-making quality of flour (Shewry et al., 2000). The rest of wheat grain proteins (about 20%) belongs to albumins and globulins, which are metabolic proteins (Žilić et al., 2011). Some wheat grain proteins, which cause human intolerances, are differentially abundant in various cultivars (Lakhneko et al., 2020).

Water restriction generally causes grain yield shortage but may increase grain protein content (Magallanes-López et al., 2017; Wan et al., 2022). So-called terminal drought (during reproductive development) is critical for grain formation and results in significant yield losses. (Farooq et al., 2014). Drought modulates albumin and gliadin content in grain rather than globulin and glutenin (Zhang et al., 2014). However, researchers reported that the content of glutenins could increase after a single early or late drought event but reduce after a single or double heat treatment (Zhang et al., 2013). Another study on hard spring wheat showed upregulation of some glutenin subunits after a terminal drought event (Labuschagne et al., 2020). Gliadin content also typically increases upon drought. However, research groups published contradictory data on whether such boost in gliadin content impacts dough quality (Gooding et al., 2003; Zhang et al., 2014). Drought stress before the end of grain filling decreased SDS-sedimentation volume compared to a water deficit applied later (Gooding et al., 2003). Some storage proteins related to bread-making quality, including α-gliadin, γ-gliadin, low molecular weight (LMW) glutenin, and globulins decreased; concurrently, one globulin and one LMW glutenin increased in abundance in response to water deficit (Yang et al., 2011).

This study aimed to comprehensively compare grain proteome profiles between drought-tolerant (Odeska 267) and drought-sensitive (Darunok Podillia) winter wheat cultivars and to determine the variable impact of drought at the flowering stage on oxidative stress and photosynthesis parameters in flag leaves. Moreover, we had the ambition to reveal potential molecular markers for the improvement of drought tolerance in bread wheat.

## 2 Materials and methods

### 2.1 Plant material and growth conditions

Odeska 267 and Darunok Podillia are commercial winter bread wheat cultivars of Ukrainian origin. These two cultivars were chosen since they originated and were recommended for cultivation in different geographical climatic zones of Ukraine. Seeds were acquired from the collection of the Institute of Plant Physiology and Genetics (Kyiv, Ukraine).

Odeska 267 is a drought-tolerant cultivar recommended for growing in Steppe and Forest Steppe zones. It belongs to the medium-sized group (110–120 cm). It is one of the least demanding varieties for growing conditions. The cultivar has high cold and drought resistance. Its heat resistance is at the level of standards for the Steppe climatic zone. The variety has high resistance to sprouting and shedding and average tolerance to laying. Grain contains 13.0–13.9% of protein. Grain wet gluten content is 28–30% and the volume of bread from 100 g of flour is 1480 mL (Catalog of cultivars and hybrids of grain, leguminous, oil, forage crops of the Plant Breeding and Genetics Institute, 2011).

Darunok Podillia is a drought-susceptible cultivar. It was registered for cultivation in the Polissia and Forest-Steppe zones of Ukraine. Plant height is 92–95 cm. This highly productive cultivar is resistant to laying and has above-average winter hardiness. It is resistant to the majority of diseases and pests, shedding, as well as germination of grain in the ear. The flour milling and bread-making indicators of the variety are good. Grain contains 14.0–14.5% protein and 28.2–31.5% wet gluten. The volume of bread from 100 g of flour is 950–1050 mL (Morgun et al., 2014).

The experiments were conducted using potted plants of two cultivars differing in their drought-tolerance: tolerant Odeska 267 and sensitive Darunok Podillia. To characterise wheat growth stages, the globally accepted scale of plant growth and development phases (phenological stages) BBCH (Biologische Bundesanstalt, Bundessortenamt und CHemische Industrie) was applied (Meier, 2018). The seeds were planted in luvisol soil (according to FAO/UNESCO Soil Map of the World, https://www.fao.org/soils-portal/data-hub/soil-maps-and-databases/faounesco-soil-map-of-the-world/en/) in the field condition in fall 2016 (100 seeds per 10 cm rows, 4 cm deep). After overwintering, seedlings were transferred to 25 cm diameter × 26 cm height pots filled with 10 kg of dry mixture of luvisol soil and sand (4:1). Nitrogen, phosphorus, and potassium were added to the pots at the rate of 160 mg per kg of soil for each element two times in equal quantities. Firstly, when the pots were filled with soil mixture and secondly, in the middle of the stem elongation period (BBCH 34—Node 4 at least 2 cm above node 3). For each cultivar, we set up 10 pots with 20 plants each. The plants were grown outdoor in pots underneath a polyethylene canopy from April. The average distance between the top of the spikes and the tent was 35 cm. The difference between the temperature outside canopy area and temperature on the level of the spikes did not exceed 1°C. The pots were watered daily to maintain the soil moisture level at 60–70% of field capacity (FC).

### 2.2 Drought treatment

Drought treatment was applied to one half of the pots at the start of the anthesis stage (BBCH 61—Beginning of flowering: first anthers visible) starting end of May. Another half of the pots were used as a well-watered control. Watering was withheld until the soil moisture reached 30% of FC. This soil moisture level was kept for 7 days, and then watering was resumed to maintain the soil moisture at the level of control plants until the harvest.

Plant performance parameters were measured on the 1^st^ day of drought at 30% of FC (the 3^rd^ day after cessation of watering) and the 7^th^ day at 30% of FC. Flag leaves were collected for measuring biochemical parameters and frozen immediately. The impact of drought on grain yield components was evaluated at harvest in July.

### 2.3 Photosynthesis and water status parameters

The physiological response of plants to drought was evaluated by measuring relative water content, chlorophyll content, CO_2_ and H_2_O exchange rates.

Relative water content (RWC) was measured as described in the literature (Bhushan et al., 2007; Cheng et al., 2016). Freshly picked flag leaves were immediately weighted to get fresh weight (FW). The leaves were incubated in distilled water in darkness at 4°C for 24 h until fully turgid to determine the turgid weight (TW). The fully turgid leaves were dried in an oven at 105°C for 24 h to determine the dry weight (DW). The RWC was calculated by the following formula: RWC (%) = [(FW – DW)/(TW – DW)] × 100.

The content of chlorophylls *a* and *b* was determined in the flag leaf by the spectrophotometric method. Pigments were extracted from crushed leaves (100 mg) with 10 mL of dimethylsulfoxide (DMSO). The test tubes with the solution were placed in a water bath for 4 h at 65°C. Then 1 mL of the solution was diluted 5 times with DMSO and the optical density was determined on a spectrophotometer SF-26 (LOMO). The concentration of pigments (mg/L) was determined according to the Wellburn equation (Wellburn, 1994), then converted to the mass of DW.

The CO_2_ assimilation, photorespiration, and transpiration rates were recorded under controlled conditions using a portable gas analyser EGM-5 (PP Systems). Flag leaves on intact plants (2 in parallel) were placed in a thermostatically controlled (25°C) chamber and illuminated (1500 μmol/m^2^·s of photosynthetically active radiation) with a TA-11 50 W LED spotlight (spectral temperature 5200 K). Conditioned air (humidity 10 mbar, CO_2_ concentration 400 ppm) was blown through the chamber at a rate of 1 L/min. The photosynthetic rate was determined in μmol CO_2_/m^2^·s, and the transpiration rate in mmol H_2_O/m^2^·s. Photorespiration rate was estimated as post-illumination CO_2_ burst during 1 min after turning off the light.

### 2.4 Oxidative stress-related parameters

Protein content in the extracts was estimated spectrophotometrically at 595 nm detecting the binding of Coomassie Brilliant Blue G-250.

The activity of superoxide dismutase (SOD; EC 1.15.1.1) was determined by the ability to inhibit the photochemical reduction of nitroblue tetrazolium (NBT). The rate of the reaction was measured spectrophotometrically at 560 nm (Giannopolitis and Ries, 1977). SOD activity was expressed in units/mg protein.

The activity of catalase (CAT; EC 1.11.1.6) was determined as the decrease of absorbance at 240 nm in µmol H_2_O_2_/mg protein·min (Aebi, 1984).

Ascorbate peroxidase (APX; EC 1.11.1.11) activity was determined by assessing the concentration of ascorbate in the presence of H_2_O_2_. The reaction rate was determined by evaluation of the absorbance at 290 nm and enzyme activity was determined in µmol ascorbate/mg protein·min (Chen and Asada, 1989).

Guaiacol peroxidase (GPX; EC 1.11.1.7) activity was evaluated as total peroxidase content in leaf extract after the release of cell wall-bound enzymes. Absorbance measurement was performed at 470 nm. Enzyme activity was expressed as μmol/mg protein·min (Vanacker et al., 2000).

The level of lipid peroxidation as malondialdehyde (MDA) equivalents was estimated by measuring thiobarbituric acid reactive substances at 532 nm. MDA content was expressed in μmol/g FW (Kumar and Knowles, 1993).

### 2.5 Yield estimation

To evaluate the productivity of two cultivars in irrigated and restricted-water conditions, grain yield components were measured: weight of grains from the whole plant and grain number from the whole plant.

### 2.6 Protein extraction

All reagents and solvents of the highest available analytical grade were purchased from Sigma-Aldrich or Merck Millipore, respectively, unless stated otherwise. Grain proteins were extracted according to the developed protocol (Dupont et al., 2011). Following the grinding of 1 g of seeds to fine flour with liquid nitrogen in a mortar, 10 mL of extraction buffer (2% SDS, 10% glycerol, 50 mM dithiothreitol, and 50 mM Tris pH 6.8) was added. The mixture was incubated with vigorous shaking for 1 h, followed by centrifugation at 10,000 × *g* for 15 min. Proteins were precipitated from the supernatant with four volumes of cold acetone and kept overnight at –20°C. The precipitate was collected by centrifugation at 4000 × *g*, 4°C for 15 min, followed by a single acetone wash. The protein precipitate was dried under a vacuum and stored at –80°C.

### 2.7 Protein separation by denaturing gel electrophoresis

Before running one-dimensional gel, protein aliquots of 20 µg each were mixed with loading buffer (60 mM Tris-HCl pH 6.8, 2% SDS, 10% glycerol, 5% β-mercaptoethanol, 0.01% bromophenol blue), and denatured at 100°C, 5 min. For protein separation, 18 cm denaturing polyacrylamide gel was prepared with 4% stacking and 13% running parts. Protein fractionation was performed in Protean II xi Cell (Bio-Rad) with running buffer (25 mM Tris, 192 mM glycine and 0.1% SDS) at 10 mA per gel for 1 h and 30 mA per gel until tracking dye disappeared, approximately 5 h. Gels were stained by sensitive colloidal Coomassie G-250. Images were digitalised with a resolution of 300 dpi and 16-bit grayscale pixel depth on Umax ImageScanner (GE Healthcare).

### 2.8 In-gel digestion

Each gel lane was manually divided into nine equal fractions (**Figure S1**), washed with 300 µL of 50 mM ammonium bicarbonate in 50% acetonitrile, and dehydrated with 300 µL of acetonitrile. Proteins were reduced with 100 µL of 10 mM dithiothreitol in 100 mM ammonium bicarbonate at 50°C for 30 min. Alkylation was performed with 100 µL of 50 mM iodoacetamide in 100 mM ammonium bicarbonate in the dark for 30 min. Gel bands were digested overnight with 100 µL of 4 ng/µL of chymotrypsin (Promega) in 10 mM ammonium bicarbonate and 10% acetonitrile at 25°C. Peptides were extracted twice with 100 µL of 70% acetonitrile and 1% trifluoroacetic acid. Subsequently, the concentration of the peptides was measured by NanoDrop 2000 spectrophotometer at 280 nm (ThermoFisher Scientific).

### 2.9 Protein identification and quantification

The peptides were analysed by liquid chromatography-tandem mass spectrometry (LC-MS/MS), using nanoAcquity UHPLC (Waters) and Q-TOF Premier (Waters). Samples were separated on BEH130 C18 analytical column (200 mm length, 75 µm diameter, 1.7 µm particle size), using a 60 min gradient of 5%–40% acetonitrile with 0.1% formic acid at a flow rate 300 nL/min. The data were recorded in the MSE mode (parallel high and low energy traces without precursor ion selection). Ions with 50–2000 m/z were detected in both channels, with a 1 s spectral acquisition scan rate.

As outlined in the literature, data processing was done in Progenesis QI 4.0 (Waters; Nováková et al., 2020). The following thresholds were applied for peak picking: low energy 320 counts and high energy 40 counts. Precursors and fragment ions were coupled using correlations of chromatographic elution profiles in low/high energy traces. Then, peak retention times were aligned across all chromatograms. Peak intensities were normalised to the median distribution of all ions, assuming the majority of signals were unaffected by experimental conditions. The label-free quantification relied on measured peak areas of the three most intense precursor peptides. Before statistical analysis, data were transformed using the inverse hyperbolic sine function. The Ion Accounting 4.0 (Waters) search algorithm was applied for protein identification. Spectra were searched against wheat proteome sequences downloaded from UniProt in April 2018 (136,892 entries, uniprot.org). Workflow parameters for the protein identification searches were: maximum two possible chymotrypsin miscleavages, a fixed carbamidomethyl cysteine, variable oxidised methionine, and deamidated glutamine. The software automatically determined the precursor and peptide fragment mass tolerances. Peptide matching was limited to less than 4% false discovery rate against the randomised database. Identities were accepted if two or more different peptides with a score higher than 95% reliability threshold were matched. Reliability scores were adjusted based on the distribution of target/decoy queries. The biological functions and technological quality features of polypeptides were taken primarily from UniProt. Obsolete accessions were replaced with analogous sequences actual for December 2022. The mass spectrometry proteomics data have been deposited to the ProteomeXchange Consortium via the PRIDE partner repository with dataset identifier PXD040279 and doi 10.6019/PXD040279 (Perez-Riverol et al., 2022).

### 2.10 Statistical analysis and visualisation

Statistical analysis of non-proteomic data was done in Prism 9.3 (GraphPad) using ANOVA followed by Tukey’s posthoc test. Proteomic data were evaluated in Perseus 1.6.15 (maxquant.net/perseus/) with the same statistical tests. Additionally, a principal component analysis was done. Next, we performed hierarchical clustering on Z-score normalised means with Euclidean distance and k-means preprocessing. Summary figure was created using BioRender.

## 3 Results and discussion

### 3.1 Water status and photosynthesis in flag leaves

To evaluate the effect of plant water status on photosynthesis, we determined the set of parameters in control and treated samples of the tolerant and the sensitive cultivar in relation to drought: RWC, transpiration rate, CO_2_ assimilation rate, photorespiration, and chlorophyll content.

**Figure 1:**
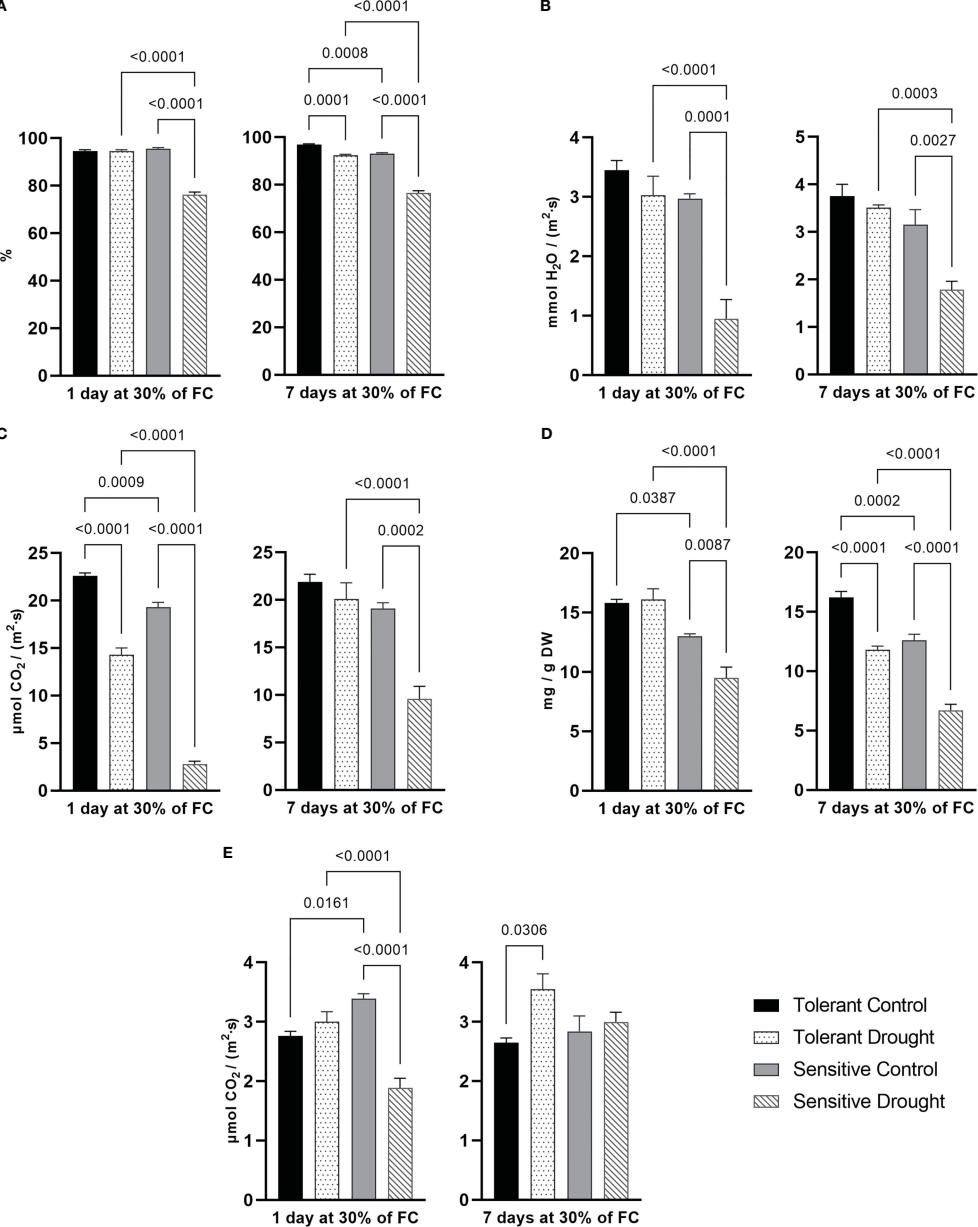
Parameters of water status and photosynthesis system measured in flag leaf at day 1 and day 7 of drought (30% of soil field capacity, FC): **(A)** relative water content; **(B)** transpiration rate; **(C)** CO_2_ assimilation rate; **(D)** total chlorophyll content; **(E)** photorespiration rate. Values are mean ± SE (*n* = 5). Significant pairs, according to Tukey’s posthoc test, are shown.

RWC directly reflects plant water status allowing to assess cell water deficit. At both experimental time points, RWC of the tolerant cultivar was not notably affected; however, RWC of the sensitive cultivar decreased by around 20% (*P* < 0.0001) in a water-restricted condition compared with respective control plants (**Figure 1A**). Transpiration rate allows to assess the rate of water loss through the leaf surface, mainly through stomata. We detected a dramatic decrease by 68% (*P* = 0.0001) between samples of sensitive cultivar after 1 day at 30% of FC. On the 7^th^ day of stress, the decrease was still substantial by 43% (*P* = 0.0027). The tolerant cultivar showed only a minor non-significant reduction of transpiration rate at both time points (**Figure 1B**). The ability to avoid water deficit in tissues is the essential marker of tolerance to drought. The tolerant genotype showed negligible change in its water status. On the opposite, the sensitive genotype exhibited dramatic water loss at both time points. The sensitive genotype decreased transpiration to improve its water-use efficiency. Several reports showed a similar pattern, linking stable RWC and tolerance of wheat to drought (Nasirzadeh et al., 2021; Qayyum et al., 2021).

The carbon dioxide assimilation rate showed a downward trend in drought conditions. Tolerant cultivar reduced it by 38% (*P* < 0.0001) at earlier time point but only slightly later. Contrastingly, the CO_2_ assimilation rate in the sensitive cultivar was remarkably lower at both time points. The sensitive cultivar reduced its photosynthetic rate by 85% (*P* < 0.0001) on day 1 and by 50% (*P* = 0.0002) on day 7 at 30% FC. On the 7^th^ day, the difference between the control and drought-treated plants decreased for both cultivars (**Figure 1C**). Drought-induced changes in chlorophyll content indirectly correspond to the level of damage to the photosynthetic system. On the 1^st^ day at 30% of FC, only the sensitive cultivar showed lower chlorophyll content by 27% (*P* = 0.0087). Nevertheless, extended drought treatment caused lowering of chlorophyll content both in the tolerant (by 27%, *P* < 0.0001) and the sensitive (by 47%, *P* < 0.0001) cultivar, yet the effect size was much more pronounced for the latter one. Of note, the tolerant genotype had overall higher chlorophyll content even in control (normal watering) conditions (**Figure 1D**). Our data suggests an explicit ability of photosynthetic apparatus of the studied wheat cultivars to adapt to prolonged moderate water deficit. This may be related to changes at the molecular level, including transcription factors regulation (Bashir et al., 2021), metabolic reprogramming (Fàbregas and Fernie, 2019), and fine-tuning of energy balancing network of photosynthesizing cells (Grieco et al., 2020; Walker et al., 2020).

Photorespiration protects photosystems from photoinhibition during stress, mainly by preventing the excessive formation of ROS when an intercellular concentration of CO_2_ is limited (Huner et al., 2002; Wada et al., 2020). In this experiment, photorespiration increased by 34% (*P* = 0.0306) at the later time point in the tolerant cultivar. On the other hand, the photorespiration rate in leaves of the sensitive cultivar decreased by 44% (*P* < 0.0001) on day 1 at 30% of FC (**Figure 1E**). Transpiration and CO_2_ assimilation rates are tightly connected with stomata conductance. Typically, avoidance of desiccation is achieved by stomata closure leading to a decline in CO_2_ intake (Bashir et al., 2021). Our data confirmed a close association between a decrease in transpiration and a significant decrease in CO_2_ assimilation rate. Higher capacity for photorespiration may be another marker of drought tolerance in wheat, such as it was characteristic for the tolerant genotype. Apparently, its delayed activation in the sensitive genotype provoked the degradation of chlorophylls.

### 3.2 Flag leaves experienced elevated oxidative stress under drought

The main cause of damage to the components of the photosynthetic apparatus under drought conditions is the formation of ROS. Antioxidative enzymes inactivate ROS and thus limit the damage (Bashir et al., 2021; Seleiman et al., 2021). Of note, numerous reports failed to demonstrate a common pattern for adaptation of the antioxidant system in water-limiting conditions. As described below and documented in the literature, we revealed that the activity of antioxidant enzymes was affected by drought to a different extent in contrasting wheat genotypes (Huseynova, 2012; Laxa et al., 2019; Nasirzadeh et al., 2021; Qayyum et al., 2021).

**Figure 2:**
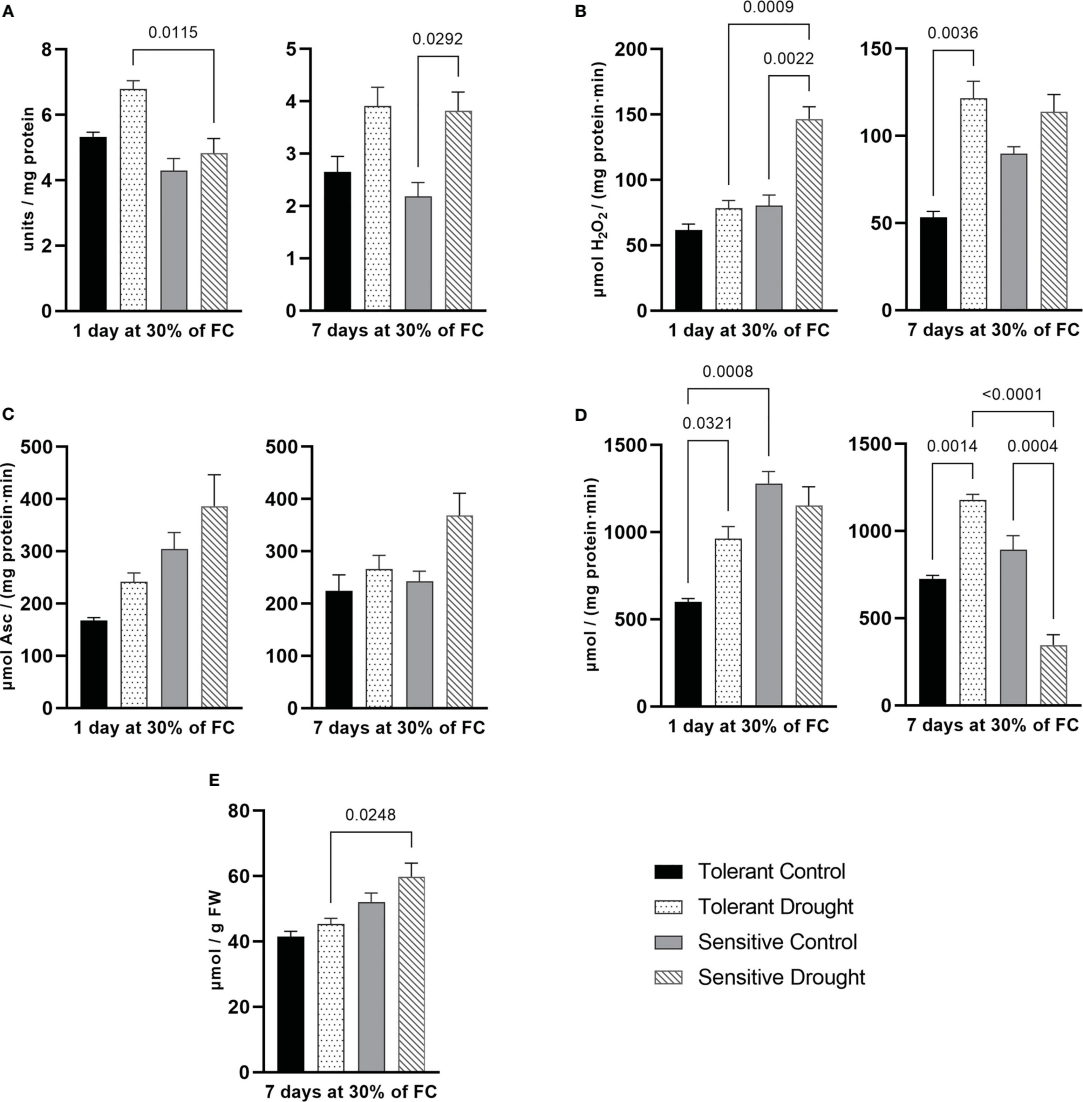
Parameters of the antioxidant system measured in flag leaf on day 1 and day 7 of drought (30% of soil field capacity, FC): **(A)** superoxide dismutase activity; **(B)** catalase activity, **(C)** ascorbate peroxidase activity; **(D)** guaiacol peroxidase activity; and **(E)** malondialdehyde content. Values are mean ± SE (*n* = 3). Significant pairs, according to Tukey’s posthoc test, are shown.

After 1 day at 30% of FC, SOD activity was about 28% (*P* = 0.052) higher in the tolerant genotype compared with the respective control. In contrast, on the 7^th^ day at 30% of FC, SOD activity boosted by 74% (*P* = 0.0292) in the sensitive and by 47% (*P* = 0.093) in the tolerant genotype. Thus, the tolerant cultivar activated SOD earlier that might lead to better protection (**Figure 2A**). Catalase behaved entirely differently: its activity was remarkably increased at early time point in the sensitive cultivar—82% (*P* = 0.0022)—yet dramatically boosted in the tolerant genotype at later time point—128% (*P* = 0.0036) (**Figure 2B**).

APX activity somewhat increased (statistically non-significantly) in both cultivars and at both time points of the experiment. After 1 day at 30% of FC, the tolerant cultivar had higher APX activity alteration (by 45%, *P* = 0.4832 versus 27%, *P* = 0.4034 in the sensitive). Prolonged drought caused 52% (*P* = 0.08) higher APX activity in the sensitive genotype compared with the respective control. Of note, the sensitive genotype had overall higher APX activity (**Figure 2C**). Activity of GPX increased in the tolerant cultivar by 61% (*P* = 0.0321) and 63% (*P* = 0.0014) on the 1^st^ and the 7^th^ day of treatment, respectively. The sensitive genotype remarkably decreased GPX activity by 61% (*P* = 0.0004) on the 7^th^ day at 30% of FC (**Figure 2D**). In essence, we detected opposite patterns for this enzyme in studied genotypes.

MDA reflects lipid peroxidation in plant tissues and its content usually increases under stress. We measured this parameter only at the later stage of drought. Its content slightly increased in the sensitive genotype (by 15%, *P* = 0.2777), although not statistically significantly (**Figure 2E**).

### 3.3 Drought stress at anthesis negatively affected grain yield

After 7 days at 30% of FC, drought-treated plants were returned to control well-watered condition at 60-70% of FC. Consequently, we measured grain yield components at harvest to evaluate the drought effect at the flowering stage on wheat performance.

**Figure 3:**
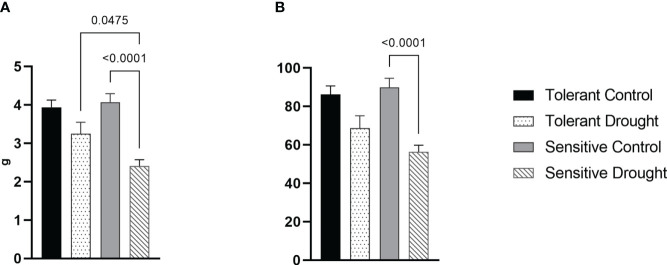
Effects of soil drought (7 days at 30% of field capacity) at the flowering stage on yield components of the winter wheat cultivars: **(A)** weight of grains from a single plant; and **(B)** number of grains on a single plant. Values are mean ± SE (*n* = 22). Significant pairs, according to Tukey’s posthoc test are shown.

The weight of grains from the whole plant was lower in both cultivars—13%, *P* = 0.1320 for the tolerant cultivar and 26%, *P* = <0.0001 for the sensitive one–compared with well-watered controls (**Figure 3A**). Similarly, grain number was somewhat lower and more substantially affected by drought in the sensitive genotype (**Figure 3B**). These measurements indicated that average seed weight was not affected by water withdrawal. Consequently, a more profound decrease of yield in the sensitive cultivar is because of a lower number of produced seeds.

### 3.4 Overview of profiled grain proteome. Proteins characteristic to genotypes

We performed grain protein profiling to determine molecular markers of tolerance/sensitivity to drought, affected by water shortage at the flowering stage of wheat ontogenesis. Protein identification and quantification details are reported in **Table S1**. We quantified 220 proteins extracted from mature grains and revealed that 142 were differentially abundant across experimental factors—genotypes and drought treatment. Principal component analysis revealed sufficient reproducibility among biological replicates. Moreover, it hinted that genotype explained a substantially higher proportion of variance than drought treatment (**Figure S2**).

**Figure 4:**
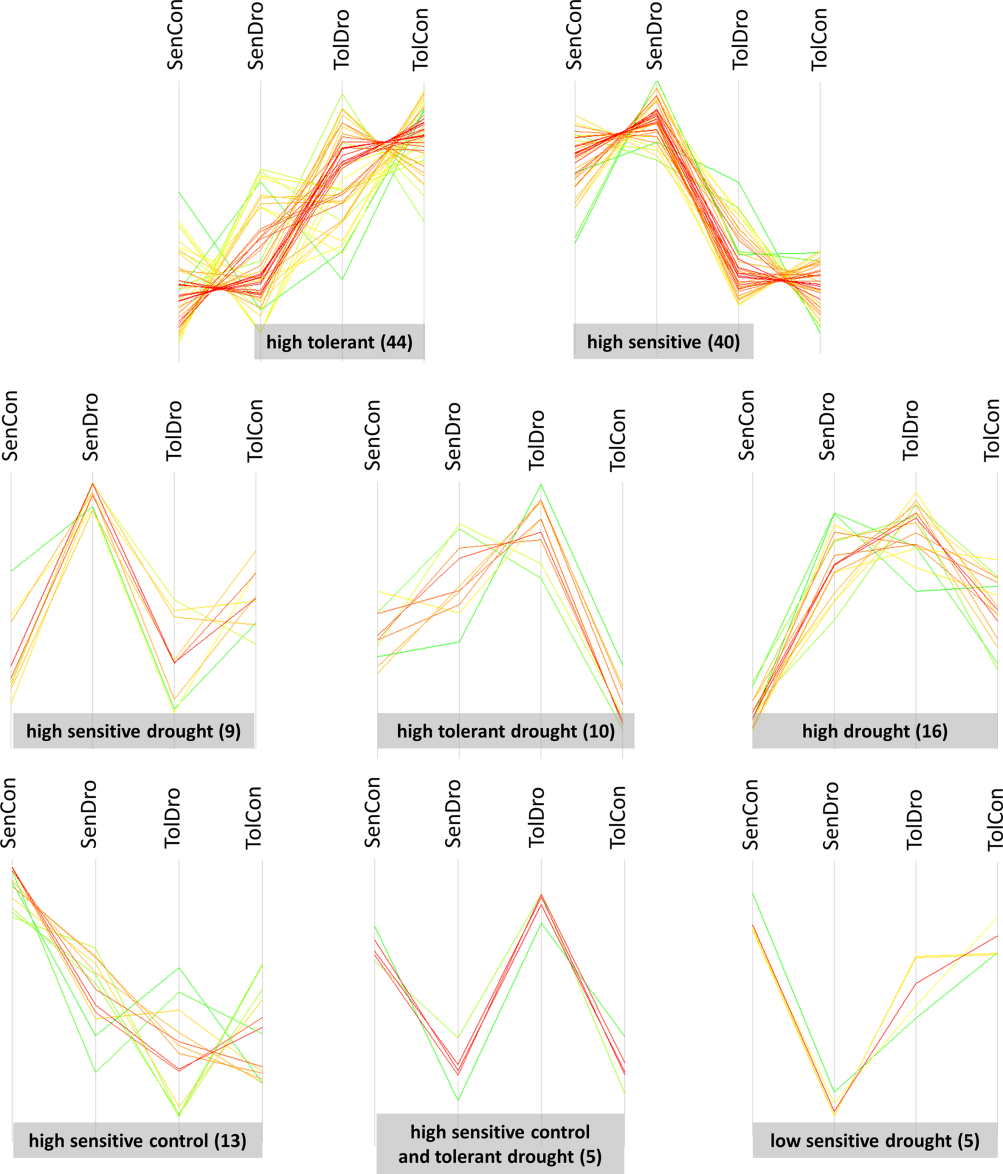
Abundance profiles within clusters of 142 differentially abundant proteins in wheat grain at harvest. Clusters were semantically named based on the similarity of accumulation profiles. SenCon, sensitive cultivar regular watering; SenDro, sensitive cultivar 7 days drought at flowering; TolCon, tolerant cultivar regular watering; TolDro, tolerant cultivar 7 days drought at flowering.

All differentially abundant proteins (ANOVA *P* < 0.05) were grouped in eight clusters: (i) ‘high tolerant’—44 identifications; (ii) ‘high sensitive’—40; (iii) ‘high drought’—16; (iv) ‘high sensitive control’—13; (v) ‘high tolerant drought’—10; (vi) ‘high sensitive drought’—9; (vii) ‘high sensitive control and tolerant drought’—5; and (viii) ‘low sensitive drought’—5. These clusters were semantically named based on the similarity of abundance change associated with a specific experimental group (**Figure 4**). Of note, genotype specific clusters—’high tolerant’ and ‘high sensitive’—are somewhat heterogenic, such as a few proteins deviate from characteristic abundance profile.

The highest number of differentially abundant grain proteins were characteristic to contrasting wheat genotypes. These proteins were clustered in two groups, ‘high tolerant’ and ‘high sensitive’, which refer to higher quantity in the respective genotype regardless conditions. Approximately half of each cluster belonged to storage proteins with nutrient reservoir activity. Thus, cluster ‘high tolerant’ contained 20 (of 44) seed storage proteins: 6 glutenins (2 high molecular weight (HMW) and 4 LMW), 11 gliadins (2 α-, 5 α/β- and 4 γ-gliadins), 2 globulins (globulin-2 and globulin-3A), and 1 avenin-like a6. The highest fold change was observed for A0A0K2QJX0 pseudo α/β-gliadin (10.9 times in the tolerant control compared with the sensitive control and 11.4 times in the tolerant drought compared with the sensitive drought), I0IT51 α/β-gliadin (7 times in the tolerant control compared with the sensitive control and 5.3 times in the tolerant drought compared with the sensitive drought), and P0CZ10 avenin-like a6 (16.2 times in the tolerant control compared with the sensitive control and 8.9 times in the tolerant drought compared with the sensitive drought).

Other grain proteins characteristic to the tolerant genotype belonged mainly to catabolic and biosynthesis pathways: two β-amylases (A0A3B6TZ67 and A0A3B6C4B2; polysaccharide catabolic process), glyceraldehyde-3-phosphate dehydrogenase (A0A3B6RKE8; glucose metabolic process), sucrose synthase (A0A3B6SD29; sucrose metabolic process), pyrroline-5-carboxylate reductase (Q5EI64; L-proline biosynthetic process), aspartic proteinase oryzasin-1 (A0A3B6PUE5; protein catabolic process). Three proteins can be related to signalling and transcriptional/post-transcriptional regulation: vinculin (A0A3B6TDU3; cell surface receptor in signalling pathway), protein argonaut 2-like (A0A3B6B4V9; RNA-mediated gene silencing), C2H2-type domain-containing protein (A0A3B6KBZ9; DNA-binding transcription factor). Three other proteins more abundant in the tolerant cultivar are associated with stress response: sHsp domain-containing protein (A0A3B6SFK5; response to hydrogen peroxide, heat and salt stress), thaumatin-like protein (Q8S4P7; defence response) and monomeric α-amylase inhibitor (X2KYP9; plant defence hydrolytic enzyme). The largest effect size was detected for DUF241 domain protein (A0A3B6EIF4; mRNA polyadenylation); it was 72.8 times more abundant in the tolerant drought than the sensitive drought and only 7.1 times higher in the tolerant control versus the sensitive control.

Similarly, cluster ‘high sensitive’ contained 22 (of 40) proteins with nutrient reservoir activity: 7 glutenins (1 HMW and 6 LMW), 9 gliadins (2 α-, 5 α/β- and 2 γ-gliadins), 3 avenin-like proteins (a4, b1 and b2), 2 AAI domain-containing proteins and 1 cupin domain-containing protein. In this subgroup, the highest differential accumulation had P04728 α/β-gliadin clone PTO-A10—7.7 times higher in the sensitive compared to the tolerant under drought and 4.3 times higher in the sensitive compared to the tolerant in the control condition. A0A3B5YPZ7 AAI domain-containing protein was about 5 times more abundant in both conditions. The effect size difference of other storage proteins varied from 1.2 to 3.6.

Another group of proteins characteristic to the sensitive cultivar united metabolic proteins: pectinesterase (A0A3B6NTI3; pectin catabolic process for cell wall modification), cytosolic triosephosphate isomerase (A0A3B6EEA7; glycerol catabolic process), and endoglucanase (A0A3B6QB34; cellulose degradation). Pectinesterase was 11.7-fold more abundant in the sensitive control than the tolerant control and 37.4-fold more abundant in the sensitive drought than the tolerant drought. Four proteins were related to transcriptional/post-transcriptional regulation: AP2-like factor (A0A3B6MP81; DNA-binding transcription factor), PB1 domain-containing protein (A0A077RVJ6; transcription regulation), SH2 domain-containing protein (A0A3B6UC79; epigenetic regulation of gene expression), RNA helicase SDE3 (A0A3B6IUP8; RNA-mediated post-transcriptional gene silencing). Another four proteins are stress- and defence-related: peroxidase (A0A077RPF9; response to oxidative stress), puroindoline-A (P33432; defence response to bacterium), chitinase (A0A3B6TZD9; defence response to fungus), and α-amylase/trypsin inhibitor CM2 (P16851; plant defence hydrolytic enzyme).

### 3.5 Proteins, which accumulated in the grain of drought-treated plants

Cluster ‘high sensitive drought’ contained 9 proteins. Among them, 8 belonged to seed storage proteins (P0CZ09 avenin-like a5, J7HT09 α-gliadin, P04724 α/β-gliadin A-IV, M9TGF7 γ-gliadin-D2, A1EHE7 γ-gliadin-D3, Q94G92 γ-gliadin-D4, A0A1D5S0Z8 11S globulin, and A0A1D5T267 cupin type-1 domain-containing protein) and only one metabolic protease—A0A3B6QML0 aspartic proteinase oryzasin-1. The effect size of accumulation of proteins with nutrient reservoir activity varied from 1.2 (M9TGF7 γ-gliadin-D2) to 1.7 (P04724 α/β-gliadin A-IV) compared with the sensitive control and from 1.1 (M9TGF7 γ-gliadin-D2) to 2.2 times (P0CZ09 avenin-like a5) compared with the tolerant drought. Aspartic proteinase oryzasin-1 was 1.6 times more abundant relative to the sensitive control and 1.3 times than the tolerant drought.

Cluster ‘high tolerant drought’ included 10 proteins: A0A3B6A1M6 AP2/ERF and B3 domain-containing protein (1.9-fold), A0A3B6FLS4 VQ domain-containing protein (1.9-fold), A0A3B6MSN0 RRM domain-containing protein (1.7-fold), A0A3B6MWJ8 SERPIN domain-containing protein (1.4-fold), Q0Q5D9 globulin 1 (1.9-fold), A0A3B6ASG7 FBD domain-containing protein (2.3-fold), Q6T484 chitinase (1.7-fold), A0A3B6QFA8 zinc ribbon 10 domain-containing protein (2.9-fold), W5BZT1 phosphatase (2.1-fold) and D0PRB4 peroxiredoxin (1.2-fold) versus the tolerant control. Accumulation of the listed proteins was less substantial in the tolerant drought relative to the sensitive drought. Specifically, only one statistically significant change—1.8-fold VQ domain-containing protein; and several non-significant ones—phosphatase (1.4-fold), FBD domain-containing protein (1.3-fold), chitinase (1.3-fold), RRM domain-containing protein (1.3-fold). In contrast to ‘high sensitive drought’, this cluster contained only a few seed storage proteins and predominantly metabolic proteins.

We detected accumulation of 16 proteins in both genotypes that experienced water withdrawal, grouped in a separate cluster ‘high drought’. Seven proteins belonged to seed storage proteins: P02861 HMW glutenin subunit PC256, B2Y2S6 LMW glutenin subunit, P16315 LMW glutenin subunit PTDUCD1, B6UKM9 γ-gliadin-A3, P06659 γ-gliadin B, M9TK56 γ-gliadin 5, and A5A4L4 avenin-like b6. Changes in the sensitive drought versus the sensitive control were more substantial than stress versus control for the tolerant cultivar. Five proteins were related to DNA binding. Two of them were A0A3B5YWA1 histone H2B and P62785 histone H4 variant TH011, which are core components of a nucleosome playing a central role in transcription regulation. Also, A0A3B6ML03, having histone deacetylation activity, plausibly negatively regulates transcription by RNA polymerase II. This protein had the largest effect size in this cluster—4.4-fold increase in the sensitive drought relative to the sensitive control, but in the tolerant cultivar remained almost unchanged (1.1-fold higher in drought versus control). Furthermore, it had genotype-specific accumulation in the control condition—4.9 times more abundant in the tolerant cultivar versus sensitive. We detected two more transcription regulators—A0A3B6RP22 transcriptional regulator SLK2 (positive and negative regulation of transcription by RNA polymerase II) and A0A3B6QFW5 BZIP transcription factor family protein (DNA-binding transcription factor activity). The former accumulated 1.3 times under stress in sensitive versus control, and the latter was about 1.7 times more abundant in both genotypes after drought compared with well-watered plants. The remaining proteins belonged to the metabolic group: A0A3B6AUV5 abhydrolase 3 domain-containing protein, A0A3B6MJZ2 5-methyltetrahydropteroyltriglutamate-homocysteine S-methyltransferase, A0A3B6I7A5 protein kinase domain-containing protein, and A0A3B6MQ45 F-box domain-containing protein. Abhydrolase 3 domain-containing protein accumulated 1.6-fold in both drought samples relative to respective controls. 5-methyltetrahydropteroyltriglutamate-homocysteine S-methyltransferase was 1.2 times more abundant in the sensitive drought versus the sensitive control and in the tolerant control versus the sensitive control. Of note, abhydrolase 3 domain-containing protein, LMW glutenin, γ-gliadin B, and γ-gliadin 5 accumulated only in the tolerant drought compared with the sensitive drought.

### 3.6 Proteins, which accumulated in the grain of the sensitive cultivar that either experienced drought or regular watering at flowering

Prominent representatives (6 of 13) of the cluster ‘high sensitive control’ were non-gluten storage proteins, such as cupin type-1 domain-containing protein (A0A3B6JER7), two globulin-1 (A0A3B6ILV9 and A0A3B6IJ76), globulin-3A (I6QQ39), and two seed biotin-containing protein SBP65-like (A0A3B5Z3G6 and A0A3B6A0K5). Cupin type-1 domain-containing protein accumulated 1.6 times compared with the tolerant control and 1.2 times than the sensitive drought. A0A3B6ILV9 globulin-1 had a similar pattern. A0A3B6IJ76 globulin-1 showed a slightly higher effect size—1.8 times compared to the tolerant control and 1.3 times compared with the sensitive drought. Seed biotin-containing protein SBP65-like accumulation was approximately 1.2 times than both the tolerant control and the sensitive drought. We detected the most substantial change for multi-pass membrane PGG domain-containing protein (A0A3B6QL90). It accumulated 3.1 times compared with the tolerant control and 2 times with the sensitive drought. We identified two α-amylase inhibitors in this cluster. α-Amylase/trypsin inhibitor CM3 (P17314) was 1.4 and 1.3 times more abundant relative to the tolerant control and the sensitive drought, respectively. This protein possesses additional serine-type endopeptidase inhibitor activity. α-Amylase inhibitor WDAI-3 (P10846) accumulated non-significantly 1.2 times versus the tolerant control and 1.1 times versus the sensitive drought.

Five proteins formed a separate cluster with higher abundance both in the sensitive control and the tolerant drought—1-Cys peroxiredoxin PER1 (Q6W8Q2; thioredoxin-dependent peroxiredoxin activity in cell redox homeostasis), 14-3-3 protein (L0GED8; signalling adaptor), chitinase (A0A3B6SU01; chitin catabolic process in defence response to fungi), SIS domain-containing protein (A0A3B6B2Y5; carbohydrate derivative metabolic process), glyceraldehyde-3-phosphate dehydrogenase (A0A3B6NQQ5; glucose metabolic process).

The cluster ‘low sensitive drought’ is particularly interesting since it includes potential markers of sensitivity to water deprivation. The most dramatic change was observed for defence response thaumatin-like protein (A0A3B6LDJ7)—20 times lower abundance than the tolerant drought and 36 times compared to the sensitive control. We also detected a change in the accumulation of one protein with nutrient reservoir activity, cupin type-1 domain-containing protein (A0A3B6ISM7), 1.3 and 1.4 times lower abundance compared to the tolerant drought and the sensitive control, respectively. Two proteins had oxidoreductase activity. The abundance of glucose and ribitol dehydrogenase (A0A3B5ZRA1) was 1.3 and 1.4 times lower than the tolerant drought and the sensitive control, respectively. Aldo keto reductase domain-containing protein (A0A3B5Z0D1) was 1.2 times statistically significantly less abundant relative to the sensitive control and 1.1 times non-significantly relative to the tolerant drought. Heat shock cognate 70 kDa protein (A0A3B6IRG6; ATP-dependent protein folding chaperone) showed the same pattern.

### 3.7 Detergent-based protein extraction contributed to the detection of differentially abundant proteins defining bread-making quality

Implementation of the SDS-based extraction protocol allows to evaluate the abundance of physicochemically diverse subgroups of wheat grain proteins (Dupont et al., 2011). The considerable proportion of differentially abundant proteins in our study were seed storage proteins, such as 44 belonged to the gluten group (gliadins and glutenins) and 6 to avenin-like proteins. A combination of glutenins (both HMW and LMW subunits) and gliadins defines bread-making qualities of dough (Barak et al., 2013). We revealed 4 HMW glutenin subunits, 12 LMW glutenin subunits, 5 α-gliadins, 11 α/β-gliadins and 12 γ-gliadins. Out of them, 36 were characteristic to genotype: 22 accumulated in the tolerant cultivar versus 14 in the sensitive genotype in well-watered conditions. Drought exposure induced differential accumulation of 31 gluten proteins. Of note, gluten fraction of grain proteome was more affected by drought in the sensitive genotype—20 differentially accumulated proteins versus 12 in the tolerant genotype. Therefore, the tolerant genotype had a more stable gluten profile, that is important for flour and dough characteristics. Interestingly, all 20 altered proteins (5 α/β-gliadins, 8 γ-gliadins, 4 HMW, and 3 LMW glutenin subunits) accumulated in the sensitive cultivar under drought. Changes were balanced in the tolerant cultivar.

Avenins are gluten-like oat storage proteins with apparently low immunoreactivity in susceptible individuals (Benoit et al., 2017; Ahola et al., 2020). Avenin-like proteins are a recently discovered group of grain storage proteins of bread wheat. Their function still remains unclear. Some of them contributed positively to flour properties (Ma et al., 2013), while others displayed antifungal activities (Zhang et al., 2018). Another recent study showed that the majority of avenin-like proteins are water- and salt-soluble, contrasting gluten proteins (Zhang et al., 2020). Furthermore, this research group revealed plenty of various domains in mature avenin-like proteins, pointing to a broad range of their functions. Researchers showed accumulation of a putative avenin a-like precursor in the endosperm under water deficit during the grain-filling stage (Gu et al., 2015). We detected changes in abundance of 6 avenin-like proteins, which were mostly characteristic to genotype—avenin-like proteins a6 and b6 accumulated in the tolerant cultivar while four others (a4, a5, b1 and b5) in the sensitive one.

### 3.8 Potential markers of drought tolerance in winter wheat

Drought caused the accumulation of 6 metabolic proteins with regulatory activities in the tolerant genotype: L0GED8 14-3-3 protein, Q6W8Q2 1-Cys peroxiredoxin, D0PRB4 peroxiredoxin, A0A077RPF9 peroxidase, A0A3B6ASG7 FBD domain-containing protein, A0A3B6A1M6 AP2/ERF and B3 domain-containing protein.

14-3-3 proteins are small and conserved adaptors which interact with their target proteins involved in hormone signalling pathways (Camoni et al., 2018). Some research groups reported their accumulation in tolerant spring wheat genotypes (Yang et al., 2011; Ge et al., 2012). Low levels of 1-Cys peroxiredoxin regulate germination and seed development in rice. Under oxidative stress, this protein gains additional function as a chaperone, active in protection (Kim et al., 2011). Besides, 1-Cys peroxiredoxin is essential for wheat grain development both in normal and water-deficit conditions (Gu et al., 2015). Spring wheat cultivar Vinjett (drought tolerance not specified) showed a lower abundance of this protein under drought, but the accumulation of its isoform under heat stress, suggesting a stress-specific role (Yang et al., 2011). Intraspecific hybridization of bread wheat with its wild relative caused the accumulation of 1-Cys peroxiredoxin in wheat line with translocation (Zou et al., 2020). Peroxidases are essential enzymatic antioxidants. We showed that one peroxidase accumulated in the grain of both the tolerant and the sensitive genotype upon water deprivation at anthesis. Previously, peroxidase accumulation was described in developing embryos under drought (Gu et al., 2015). FBD domain-containing protein does not have a well-defined known function. Researchers proposed its nuclear localization and plausible role in activation of transcription (Doerks et al., 2002). AP2/ERF and B3 domain-containing protein includes two functional domains, plausibly associating it with transcriptional regulators (Suzuki et al., 1997; Xie et al., 2019).

In our study, the sensitive genotype showed differential amount of 8 potentially regulatory proteins upon water deficit—6 accumulated (A0A3B6KUX1 HSF domain-containing protein, A0A3B6B4V9 protein argonaute 2-like, A0A3B6SFK5 sHsp domain-containing protein, A0A3B6UC79 SH2 domain-containing protein, A0A3B6RP22 transcriptional regulator SLK2, and A0A3B6ML03 member of histone deacetylase complex) and 2 less abundant (A0A3B6LDJ7 thaumatin-like protein and A0A3B6IRG6 heat shock cognate 70 kDa protein). Recently the prominent role of thaumatin-like proteins was confirmed in wheat exposed to multiple abiotic stresses (Djemal et al., 2022; Sharma et al., 2022). Heat shock cognate 70 kDa proteins perform a crucial role both in the plant life cycle and in stress responses. Published data suggested that deficiency of heat shock cognate 70 kDa protein determined the sensitivity of plant genotype to osmotic stress, such as its upregulation ensured enhanced tolerance (Ding et al., 2022). Members of the small heat shock protein (sHsp) family are highly reactive in plants under stresses but also during normal growth. They act as chaperones protecting cellular components from irreversible heat stress-induced changes (Siddique et al., 2008). We detected not only a significant accumulation of sHsp domain-containing protein in the stressed sensitive genotype compared with the respective control, but also the tolerant cultivar showed a higher abundance of this protein compared with the sensitive genotype. HSF (heat shock factor) domain-containing protein belongs to a broad protein family. Some HSFs were expressed in the wheat endosperm in normal conditions, while others were upregulated under abiotic stresses, including water deprivation (Xue et al., 2014). Furthermore, researchers proved that overexpression of *TaHsfA6f* leads to increased tolerance to multiple abiotic stresses (Bi et al., 2020). Four other proteins, which accumulated in the sensitive genotype upon drought (protein argonaute 2-like, SH2 domain-containing protein, transcriptional regulator SLK2, and member of histone deacetylase complex) are putatively involved in transcriptional and post-transcriptional regulation but specific experimental data are missing.

Cupin proteins comprise a large superfamily of plant proteins with broad functionality (Khuri et al., 2001). Analysis of some cupin domain containing globulins revealed their expression during wheat grain maturation under normal condition (Altenbach et al., 2009). Cupins accumulation can be affected by biotic and abiotic stresses, including heat and chronic ionising radiation (Gábrišová et al., 2016; Tomás et al., 2022). In our dataset, 6 cupin type-1 domain-containing proteins were differentially accumulated, including 11S globulin, two globulin-1, globulin-2, two globulin-3A. Upon drought, 4 cupin type-1 domain-containing proteins were less abundant and one accumulated in the sensitive genotype against its control. The tolerant cultivar had only one less abundant cupin type-1 domain-containing protein upon water deficit. Such variable abundance pattern of cupin type-1 domain-containing proteins likely mirrors a stronger drought impact on the sensitive cultivar than the tolerant.

### 3.9 Drought-mediated accumulation of allergenic proteins was less pronounced than genotype association

Products of inefficient digestion of gluten proteins can affect the health of sensitive individuals provoking specific diseases (respiratory allergy, food allergy, and celiac disease) (Scherf et al., 2016).

Of note, 20 of 42 differentially abundant gluten proteins are annotated as potential allergens in UniProt and Allergome (https://www.allergome.org/) databases. Six α/β-gliadins were classified based on sequence similarity; 7 LMW glutenin subunits were allergen Tri a 36 (Allergome code 2674); 3 HMW glutenin subunits were allergen Tri a 26 (code 2898); and 4 γ-gliadins were allergen Tri a 20 (code 3678). Most of them (17 identifications) had genotype-specific accumulation patterns in well-watered conditions—9 proteins were more abundant in the sensitive and 8 in the tolerant genotype. In water-restricted conditions, the sensitive genotype accumulated 8 allergenic proteins (3 HMW glutenins, 1 LMW glutenin, 2 α/β-gliadins, and 2 γ-gliadins). In contrast, the tolerant cultivar showed a higher abundance of only 3 immunoreactive LMW glutenins and a lower amount of a single α/β-gliadin.

α-Amylase/trypsin inhibitors are storage proteins of most cereals and belong to albumins (water-soluble fraction). They possess inhibitor activity against alien digestive enzymes, thus protecting plants from pests and pathogens. Their inhibitor activities can trigger allergenic response and immune intolerance in susceptible individuals; however, they are considered minor triggers (Geisslitz et al., 2022). We detected a different abundance of α-amylase/trypsin inhibitors CM2 and CM3 (P16851 and P17314) upon water deficit, while both have potential immunoreactivity according to Allergome: α-amylase/trypsin inhibitor CM2 contains Tri a 29 epitope (code 8188) and α-amylase/trypsin inhibitor CM3 had Tri a 30 and Tri a 30.0101 epitopes (codes 1051 and 8191). In control, these proteins were more abundant in the sensitive cultivar compared with the tolerant one. In the water deficit condition, the sensitive cultivar lowered the amount of α-amylase/trypsin inhibitor CM3, having the same amount as the tolerant drought, while the tolerant drought had a slight non-significant increase compared with the respective control. α-Amylase/trypsin inhibitor CM2 showed an opposite accumulation pattern upon drought. Amount of α-amylase/trypsin inhibitor CM3 in spring wheat cultivar Vinjett decreased under drought (Yang et al., 2011). Several studies on contrasting genotypes reported lower accumulation of α-amylase/trypsin inhibitor in sensitive cultivars and abundance in the tolerant ones under stress (Altenbach, 2012; Jiang et al., 2012). This is very well in line with our data.

Chitinases degrade chitin and are newly reported sources of food allergy with high reactivity (Šotkovský et al., 2011). There is no data in the literature pointing to the accumulation of immunoreactive chitinases in wheat grains under drought, yet moderately higher amount was detected under heat stress (Altenbach, 2012). Another report showed a higher abundance of class II chitinase in developing grain of multi-resistant cultivar Ningchun 4 under water deficit (Ge et al., 2012). Accumulation of class II chitinase was described under drought both in the embryo and endosperm of the Chinese winter wheat cultivar Jing 411 with broad adaptability (Gu et al., 2015). Of note, these chitinases do not have proven allergenic properties. Our data showed that upon water deprivation, both genotypes accumulated Q6T484 chitinase, which is deposited in Allergome database under the name Tri a Endochitinase (code 9595). The tolerant cultivar had a more substantial accumulation of this protein.

Peroxiredoxins are a type of peroxidases that are expressed mainly in the nucleus of aleurone and embryo in seeds suffering oxidative stress (Pulido et al., 2009). Surprisingly, the reactivity of 1-Cys peroxiredoxin (PER1) was described in relation to baker’s asthma and general food allergy (Sander et al., 2011; Lupi et al., 2013). Our data showed differential accumulation of two peroxiredoxins with allergenic epitopes registered in Allergome database as Tri a 32 and Tri a 32.0101 (codes 6327 and 9499). Both detected peroxiredoxins were accumulated in the tolerant cultivar after drought. In well-watered conditions, the tolerant genotype had non-significantly higher amount of these proteins relative to the sensitive cultivar. Other researchers showed different patterns of peroxiredoxin accumulation in different genotypes under drought. Higher abundance was detected both in the embryo and endosperm of the Chinese winter wheat cultivar Jing 411 (Gu et al., 2015). Another research group showed a lower amount of this protein in the spring wheat cultivar Vinjett (Yang et al., 2011).

Summarizing, despite a nearly equal contribution from both genotypes in the number of differentially accumulated allergenic gluten proteins in well-watered conditions, the sensitive cultivar showed considerably more medically-relevant proteins accumulated after drought. Just opposite, water restriction caused a higher abundance of allergenic metabolic proteins (chitinase and peroxiredoxins) in the tolerant cultivar. Additionally, the sensitive genotype had a lower abundance of α-amylase/trypsin inhibitors.

### 3.10 Future prospects and wheat improvement strategies

Current breeding programs aim to combine the majority of positive traits in one variety, thereby reducing the genetic diversity of modern varieties and reducing plasticity to adapt to variable environmental conditions. Recent tendency points to the introgression of genetic resources into bread wheat from its wild relatives. For example, studies proved the importance of interspecific hybridization for the supplementation of the genetic pool of modern commercial cultivars with drought-resistant proteins (Zou et al., 2020; Lu et al., 2022). The stability of grain quality under a water deficit is an essential characteristic of a cultivar. Consequently, one of the key targets for modern breeders is the development of tolerant bread wheat genotypes (Cattivelli et al., 2008; Chapman et al., 2012; Daryanto et al., 2017). Wheat may be improved by focusing on enhancing breeding strategy by the introduction of diverse sources of germplasm into crossing programs (Keser et al., 2017; Lakhneko et al., 2021). Researchers suggested to maintain the stability of the wheat grain composition under drought by interspecific hybridisation with its wild relatives from the genus *Aegilops* (Rakszegi et al., 2019). Storage protein content can be adjusted by regulating microRNAs expression, which are plausibly involved in the biosynthesis of storage proteins (Chen et al., 2017). Of note, early flowering is a viable strategy for drought escape, thus beneficial for advanced cultivars growing in arid areas (Shavrukov et al., 2017; Frantová et al., 2022). Proteome changes might reveal promising genetic markers and, consequently, facilitate marker-assisted breeding (Altenbach, 2012; Ghatak et al., 2017).

**Figure 5:**
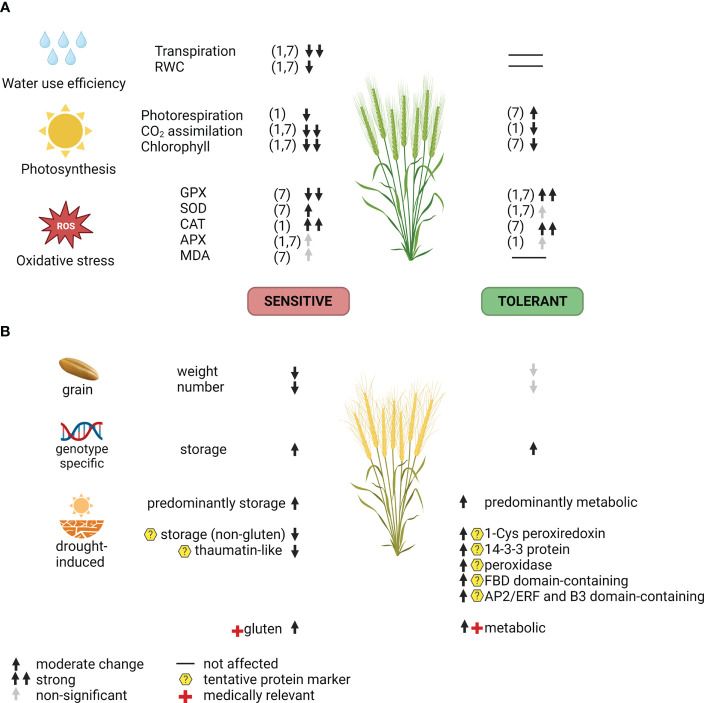
Summarized response of the sensitive and the tolerant wheat cultivar to drought at the flowering stage on **(A)** flag leaves biochemical/physiological level and **(B)** grain yield and proteome. Numbers 1 and 7 indicate a day at 30% of field capacity. MDA, malondialdehyde; APX, ascorbate peroxidase; CAT, catalase; SOD, superoxide dismutase; GPX, guaiacol peroxidase; RWC, relative water content; ROS, reactive oxygen species.

The present study analysed flag leaf physiology (**Figure 5A**) and grain proteome (**Figure 5B**) in tolerant (Odeska 267) and sensitive (Darunok Podillia) bread winter wheat cultivars exposed to drought. Proteomic profiling revealed a more stable composition of grain proteins with nutrient reservoir activities in the tolerant genotype accompanied by a lower magnitude of differential accumulation of allergenic proteins. Additionally, we proposed potential markers for drought tolerance: peroxiredoxin, thaumatin-like protein, peroxidase, 14-3-3 protein, FBD domain-containing protein, and AP2/ERF plus B3 domain-containing protein. They should be screened on multiple wheat genotypes using targeted assays, such as immunodetection or targeted mass spectrometry.

## Data availability statement

The datasets presented in this study can be found in online repositories. The names of the repository/repositories and accession number(s) can be found below: ProteomeXchange Consortium - PXD040279.

## Author contributions

Conceptualisation, OS and OL; methodology, OL, O-SS, DK, MK and OS; formal analysis, OL, OS and MD; investigation, OL and MD; data curation, MD; writing – original draft preparation, OL and MD; writing – review and editing OS and ĽŠ; visualisation, OL and MD; supervision, MD and ĽŠ; funding acquisition OL, MD and OS. All authors contributed to the article and approved the submitted version.
