# Supplementary material for: Transient drought during flowering modifies the grain proteome of bread winter wheat
Source: Front Plant Sci. 2023 Jun 27;14:1181834. doi: 10.3389/fpls.2023.1181834 (PMC10333505; doi:10.3389/fpls.2023.1181834)
Supplement: Supplementary file 1 [file DataSheet_1.docx]

Supplementary Material

Transient drought during flowering modifies the grain proteome of bread winter wheat

Olha Lakhneko^1,2^, Oleg Stasik^3^, Ľudovit Škultéty^4^, Dmytro Kiriziy^3^, Oksana Sokolovska-Sergiienko^3^, Mariia Kovalenko^5^, Maksym Danchenko^2*^

^1^Institute of Cell Biology and Genetic Engineering, National Academy of Sciences of Ukraine, Kyiv, Ukraine

^2^Institute of Plant Genetics and Biotechnology, Plant Science Biodiversity Centre, Slovak Academy of Sciences, Nitra, Slovak Republic

^3^Institute of Plant Physiology and Genetics, National Academy of Sciences of Ukraine, Kyiv, Ukraine

^4^Institute of Virology, Biomedical Research Centre, Slovak Academy of Sciences, Bratislava, Slovak Republic

^5^ESC “Institute of Biology and Medicine”, Taras Shevchenko National University of Kyiv, Kyiv, Ukraine

***Correspondence:**Maksym Danchenko
maksym.danchenko@savba.sk

**
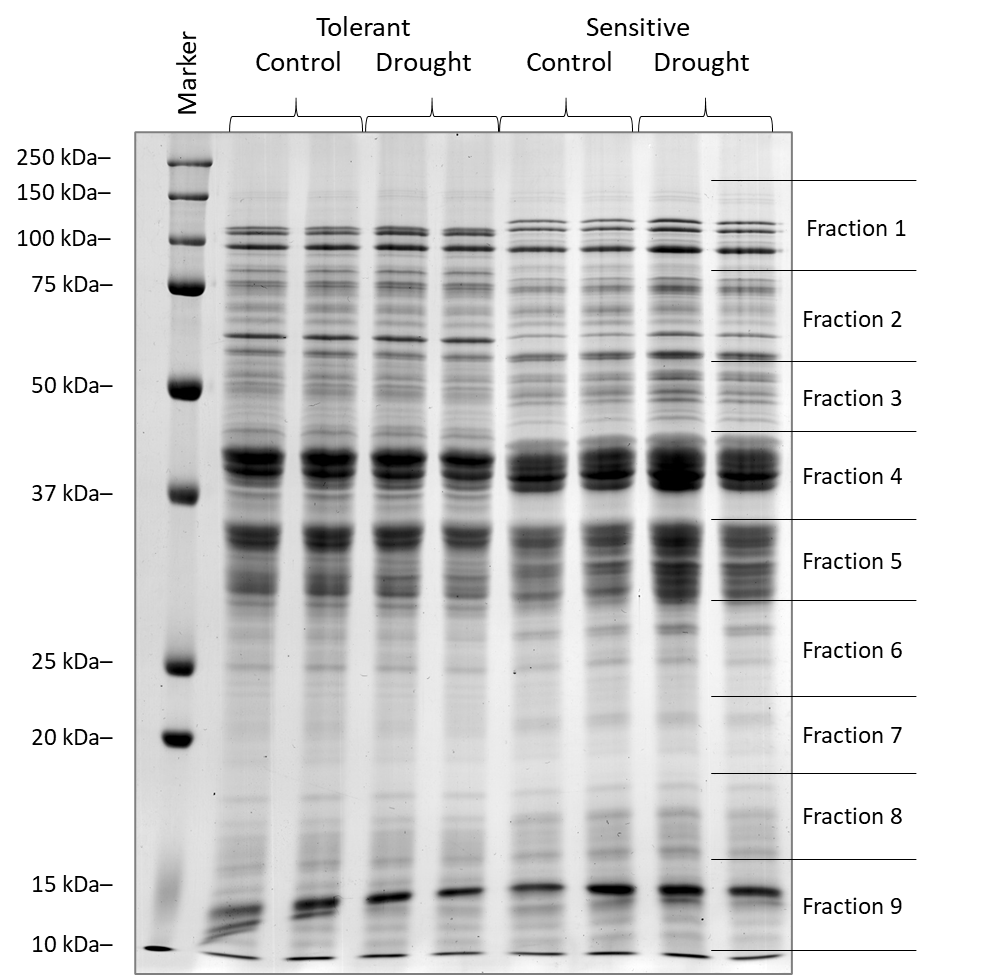
Supplementary Figure 1.** Image of representative gel stained by Colloidal Coomassie. Grain proteome extracts were profiled by denaturing gel electrophoresis according to molecular weight and separated into nine equally sized fractions.


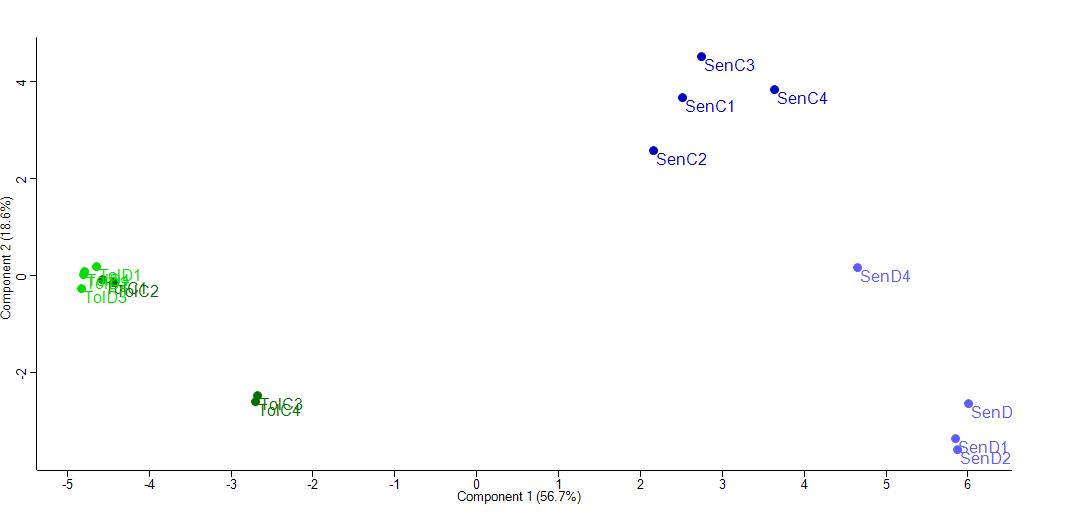


**Supplementary Figure 2.** Principal component analysis of proteins differentially accumulated in grains of tolerant cultivar Odeska 267 (Tol) and sensitive cultivar Darunok Podillia (Sen) grown in well-watered 60% field capacity (C) and water deficit 30% field capacity for 7 days (D) at anthesis.
